# ISSR molecular markers and anatomical structures can assist in rapid and directional screening of cold-tolerant seedling mutants of medicinal and ornamental plant in *Plumbago indica* L.

**DOI:** 10.3389/fpls.2023.1149669

**Published:** 2023-07-03

**Authors:** Yirui Li, Xu Cheng, Junlin Lai, Yunzhu Zhou, Ting Lei, Lijuan Yang, Jiani Li, Xiaofang Yu, Suping Gao

**Affiliations:** College of Landscape Architecture, Sichuan Agricultural University, Chengdu, China

**Keywords:** combined tissue culture and chemical mutagenesis techniques, ISSR analysis, anatomical structure, targeted screening for cold-resistant mutants, cold resistance evaluation

## Abstract

*Plumbago indica* L. is a perennial herb with ornamental and anticancer medicinal functions widely distributed in the tropics. It is affected by temperature and cannot bloom normally in colder subtropical regions, which seriously affects its ornamental value. To create low-temperature resistance mutants and enrich new germplasm resources, this study used tissue culture and chemical reagent (0.5 mmol/L NaN_3_) and low-temperature stress (0°C, full darkness for 48h) induction to target and screen for cold-resistance mutants. The results showed that the ISSR band polymorphism ratio of the 24 suspected mutant materials was 87.5%. The DNA profiles of the 9 mutants initially identified were altered. The content of plumbagin in the stems and leaves of the mutants was examined, and it was found that the accumulation in the leaves of the mutant SA24 could be as high as 3.84 times that of the control, which was 0.5991%. There were significant differences in the anatomical structures of roots, stems and leaves. The mutants mostly exhibited reduced root diameter (only 0.17-0.69 times that of CK), increased stem diameter (up to 2.19 times that of CK), enlarged mesophyll cells, increased thickness (up to 1.83 times that of CK) and high specificity, which are thought to be important for the different cold resistance obtained by the mutants. In the cold resistance experiment, four cold-tolerant mutants were successfully screened according to their morphological characteristics and physiological indexes, and the mutagenesis efficiency could be as high as 2.22% and did not affect the accumulation of plumbagin in their stems and leaves, even higher than CK. The responses of the screened mutants SA15, SA19, SA23 and SA24 to low temperature showed slower leaf wilting, higher light energy conversion efficiency, less accumulation of MDA content, increased enzymatic activities of antioxidant enzymes (SOD, CAT, POD) and more accumulation of soluble sugars and proline content. These characteristics are consistent with the response of cold-resistance plants to low temperatures. The cold- resistance mutants cultivated in soil were observed of agronomic and ornamental traits for one year, mainly manifested as delayed flowering and delayed entry into the senescence stage. This study provides a more rapid and accurate technique for identifying and screening cold-tolerant mutants, and lays the foundation for future experiments on the creation of new cold-resistant varieties.

## 1 Introduction

Cold is one of the major environmental stresses that limit plant growth and development, affects geographic distribution, and significantly reduces plant biomass. The increasing frequency and severity of extreme weather and climate events are one of the most pressing threats facing humanity today (Robinson, 2021; Liu et al., 2022). Extreme environmental changes have led to serious threats to the survival and growth of ornamentals introduced and cultivated from higher heat locations (Zheng et al., 2022). These plants are usually subjected to cold stress at temperatures below 10°C, or even higher (Zhuang et al., 2021).Cold damage leads to complex changes in plant morphology, physiology and biochemistry, which trigger complex cellular tissue dysfunction in plants (Knight and Knight, 2012), such as epidermal tissue damage or even necrosis in plant organs (Chen et al., 2020b). Plants establish cold acclimation in order to overcome the stress caused by exposure to freezing-injurious environments through complex biological system regulation that induces changes affecting biomass such as lignin in nutrient organs such as rhizomes (Podgorbunskikh et al., 2020), as well as changes in photosynthesis, enzyme activity, carbon metabolism, and protein metabolism *in vivo *(Theocharis et al., 2012).

**Figure 1:**
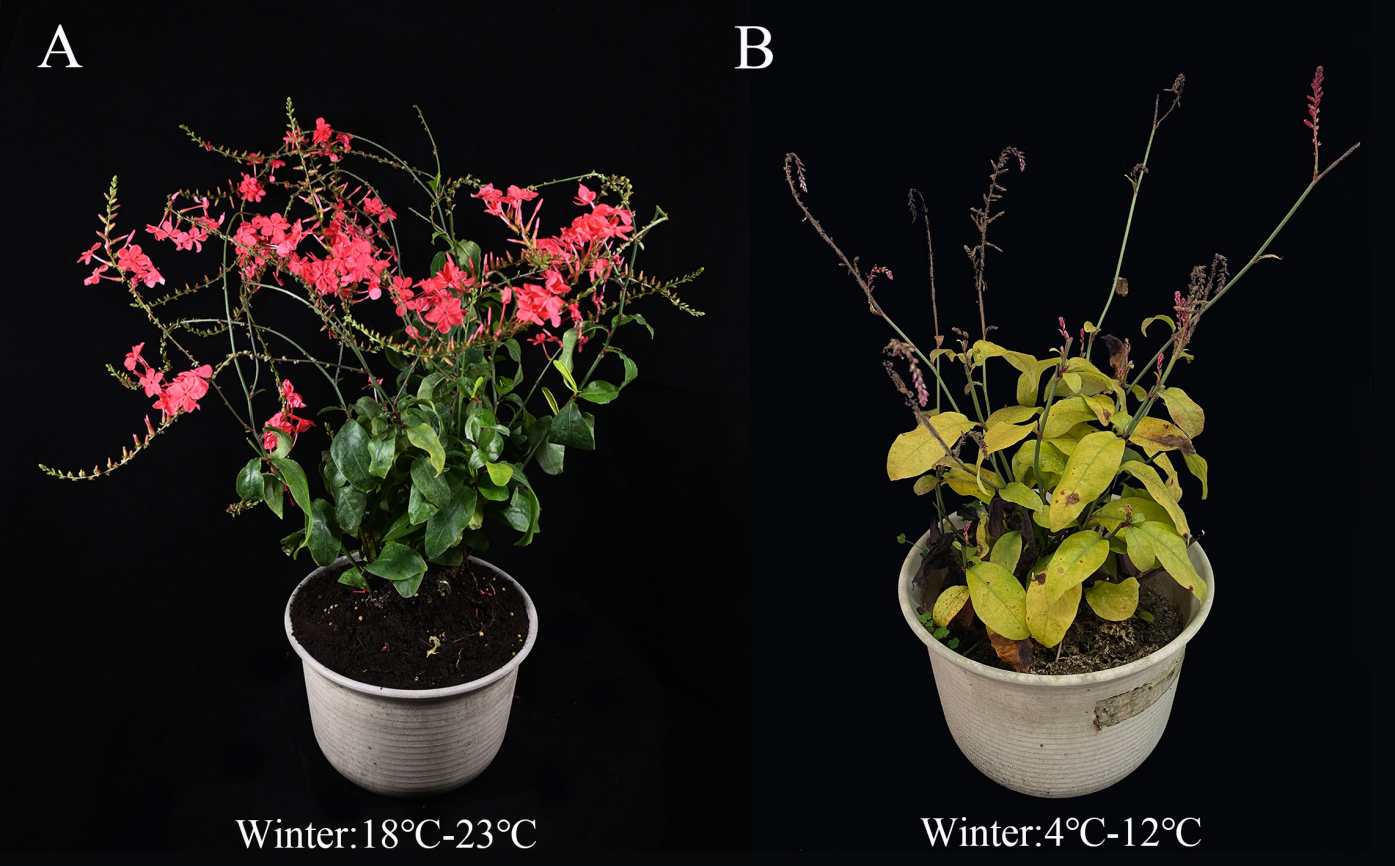
Comparison diagram of growth status of *Plumbago indica* L. wild planted in Hainan province and Sichuan province from December to February (winter average temperature). **(A)**, cultivated in Hainan province; **(B)**, cultivated in Sichuan province.

*Plumbago indica* L. is a tropical evergreen perennial plant of the genus Plumbago in the family Plumbaginaceae. In China, it is native to areas such as Yunnan (Hekou), Guangdong and Hainan provinces, where heat accumulation is high. Its corolla is purplish red or crimson and flowers from November to April (in some places it blooms all year round). Its root secondary metabolite, plumbagin, is also utilized as an anticancer (Li et al., 2014; Vasav et al., 2020) and antibacterial (Venkateswarulu et al., 2018) drug ingredient. This is introduced to the subtropical region (Chengdu region, for example), and the temperature starts to drop in November every year. Although the flower buds can be differentiated normally and some branches can form flakes, the low temperature at the time of flowering has a serious impact on the complete unfolding of the petals, resulting in a serious shortening of the flowering period and a major loss of ornamental value (**Figure 1**). Therefore, improving its cold tolerance is an important goal for breeding. Since *Plumbago indica* L. is a heterostylous plant and has not been seen to bear fruit in nature, it is difficult to improve its cold hardiness by cross breeding. Integrated tissue culture and chemical mutagenesis for mutant induction provides a new breeding technique for creating new cold-tolerant mutant materials that can shorten breeding cycles and accelerate breeding programs (Shi et al., 2022) and has been successfully applied to several plants, including rice (Chen et al., 2013), *Cerastigma willmottianum* Stapf (Shi et al., 2022), olive (Sheikh and Moradnejad, 2014).

Traditional plant breeding utilizes natural mutation that can randomly produce a number of variant traits, and since the discovery of mutagens, various mutagenesis methods have been widely used in plant molecular breeding to identify new traits conferred to plants (Shelake et al., 2019). The use of DNA technology, mutation breeding to create new varieties in response to abiotic stresses has become a popular research theme (Janni et al., 2020). Among them, the effect of chemical mutagens can be detected by morphological characteristics, genetic mapping and changes in chromosome number (Chaudhary et al., 2019). Traditionally morphological character changes can be used to screen for mutants. However, this method is both time-consuming and unreliable due to environmental and multiple factors. In addition, mutations in many plant genes may not result in readily identifiable phenotypes. Molecular markers have been used for genetic identification and improvement of many crop species. Recently, molecular breeding and traditional breeding techniques have been used to obtain new varieties (Gentile and La Malfa, 2019). Therefore, direct selection based on DNA markers circumvents these limitations, thereby increasing efficiency and reducing the cost and time of selection.

Molecular marker technologies have different approaches based on different principles, but all contribute to the successful identification of variation loci in the whole genome (Polat et al., 2015). Among them, ISSR has been widely used to assess plant genetic diversity, genetic stability, and the identification and screening of mutant plants (Wu et al., 2020). The advantage of this technique is that the designed microsatellite sequences are randomly distributed in the plant, deletions/duplications at binding sites can be used with ISSR primers, and multiple motifs can be detected simultaneously with high reliability and reproducibility (Li et al., 2018a), making it particularly suitable for the analysis of many mutants with limited genetic variation. This technique has been used previously in the screening of mutants in several ornamental plants, such as tulips (Li et al., 2022c), chrysanthemums (Wu et al., 2020), and has also worked well. Therefore, the present study uses ISSR-PCR markers combined with anatomical structural features of nutrient organs to identify mutant ideas, which is a combination of microscopic and macroscopic identification tools, and provides a practical technical solution for the identification of new mutant materials created by tissue culture and chemical mutagenesis (NaN_3_) in *Plumbago indica* L. Meanwhile, artificial low temperature stress experiments using plant tissue culture ex vivo platform provide a new pathway for rapid screening of cold-tolerant mutant strains for early targeted breeding.

## 2 Materials and methods

### 2.1 Plant materials

**Figure 2:**
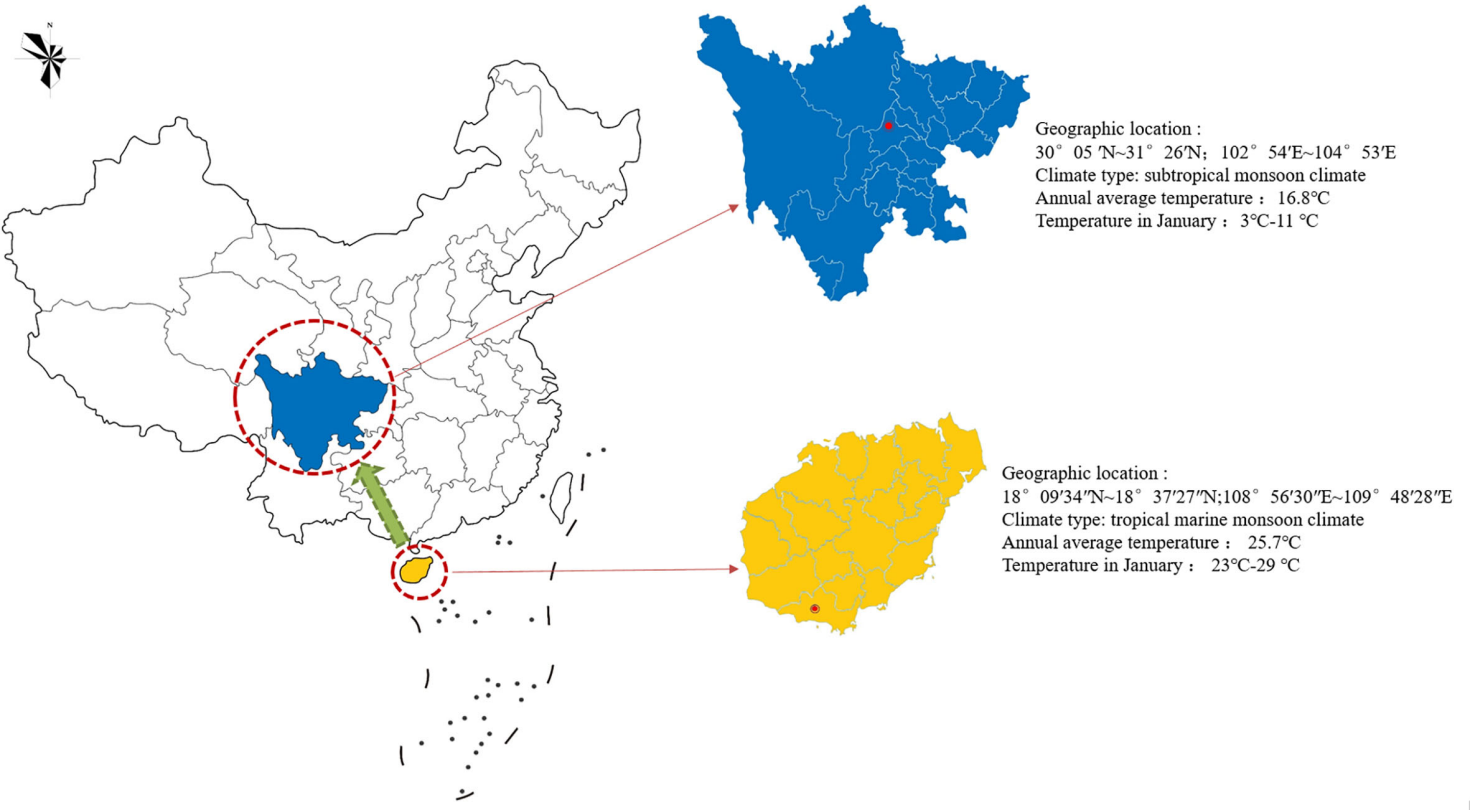
Geographical tagging of the research area in China. The red circle indicates the introduction or cultivation site of *Plumbago indica* L. in China, and the green arrow indicates the introduction and cultivation direction in China.

The experimental materials were cultivated species in Sanya, Hainan Province. After being introduced and cultivated from Chengdu, Sichuan Province, sterile seedlings were cultivated for mutation breeding (**Figure 2**). These were 26 plants 90 d tissue culture seedlings of the same genotype, which contained 24 suspected mutant plants and 2 wild control plants. The 24 suspected mutants were sourced from: A single-factor experiment was conducted to mutagenize the terminal buds of sterile seedlings of *Plumbago indica* L. using NaN_3_ as mutagen. And it was set at 0 mmol/L, 0.1 mmol/L, and 0.2 mmol/L, 0.3 mmol/L, 0.4 mmol/L, 0.5 mmol/L, 0.6 mmol/L, 0.7 mmol/L, 0.8 mmol/L, 0.9 mmol/L; 0h, 0.5h, 1h, 2h, 4h, 8h; pH=3.0, NaN_3_ concentration was referenced Sen (2016) and Szurman-Zubrzycka et al. (2018); single-factor experiments were used to mutate the terminal buds of *Plumbago indica* L. sterile seedlings (30 as a group, biological replicated 3 times), and the survival rate was counted after 20 d. The LD_50_ was used as the best mutagenic formulation of NaN_3_. Based on the pretreatment results, 180 sterile terminal buds (VM_0_) from the same mother plant (60 in one group, biological replicates three times) were finally treated with *in vitro* chemical mutagenesis using the best NaN_3_ mutagenesis formulation (0.5 mmol/L, 1 h, pH=3.0). The terminal buds of VM_1_ were inoculated and succeeded as VM_2_. And so on, successive successions were cultured for 5 generations to VM_5_. Rooted plants were counted under environmental conditions of 25 ± 1°C, 12 h/d photoperiod, and 1500-2000 lx light intensity. After 30d acclimatization, the seedlings were transplanted to pots and cultivated for 90d, and plant morphological variation was observed and evaluated in the field. Plants with morphological variation rate greater than 25% were used as suspected mutant plants for this experiment, and a total of 24 suspected mutant plants were obtained. Two controls were wild-type plants without NaN_3_ treatment, one treated with sterile water set as CK and one treated with phosphate buffer solution set as PBS.

### 2.2 ISSR molecular markers and analysis

The CTAB method was used to extract the total DNA from the young leaves of CK, PBS and 24 suspected mutant plants, respectively, and the extracted DNA was stored in a refrigerator at -20°C for backup. Synthesis of 100 universal primers (Sangon Biotech, Shanghai) published by UBC (University of British Columbia) for mutant plant screening. The optimized 25 μL ISSR-PCR amplification system (Li et al., 2022c) was 12.5 μL 2xPCR Master Mix, 1 μL template DNA, 1 μL primer, and 10.5 μL ddH2O. The PCR reaction system was pre-denatured at 95°C for 3 min, denatured at 95°C for 15 s, annealed at 61°C for 15 s, extended at 72°C for 1 min, 32 cycles, and then extended at 72°C for 5 min to complete the amplification and terminate the program at 12°C. The obtained products were used for gel electrophoresis experiments. The gel was evaluated on a 1.5% agarose gel with 10 μL TS-GelRed Ver.2 Nuclear Staining Dyes and 8 μL product, and the plate was run in 1× TAE buffer at 150 V for 20 min. After completing electrophoresis, pictures were taken and analyzed on a gel imaging system.

### 2.3 Plumbagin content of medicinal ingredients in stems and leaves by HPLC

9 plants from each of the CK and nine mutant strains (three plants per group in three biological replicates) were transplanted for 60 d, and fresh samples of stems and leaves were collected separately and stored in liquid nitrogen for freezing. The cryopreservation samples were dried at 65°C for 24h, followed by adjusting the temperature to 45°C and drying to constant weight, and then ground into powder for later using. Precisely weigh 0.10 g of sample powder, extract with 1 mL of methanol (HPLC, purity ≥ 99.9%) at ordinary temperature for 30 min by ultrasonication, collect the extract and filter using 0.22 μm microporous filter (Li et al., 2022c), and store the filtered extract at 4°C away from light. The analysis of plumbagin was performed by LabSolitions-PAD system. Experimental reference Nayak et al. (2015) experimental method, and the method was optimized the sample injection volumes were all 20 μL, and the separation was performed on a Shim-pack GIS C18 (4.6×250 mm, 5 μm) column at a flow rate of 1mL/min and a column temperature of 35°C. The mobile phase A was methanol and liquid B was 0.1% H_3_PO_4_ (vacuum filtered through a 0.45 μm filter membrane before use). The gradient elution steps were: 40% A, 60% B, 0 min; 75% A, 25% B, 21 min; 90% A, 10% B, 23 min; 90% A, 19% B, 30 min; 40% A, 60% B, 40 min. Plumbagin was detected at 254 nm. A linear regression equation *y*=8.20712×10^7^x+151720.60002 (*R*_2_ = 0.98543) was constructed for the quantitative analysis using plumbagin standard (CAS: 481-42-5, Solarbio, purity≥98%).

### 2.4 Analysis of cellular anatomy

At 9:00 a.m., 26 (including 2 controls) plants of 120d seedlings after acclimatization cultivation were taken for cytological observation of roots (main root maturation zone about 1 cm long), stems (about 1 cm long) and leaves (1 cm wide and 2 cm long). Samples were taken and fixed individually in microcentrifuge tubes containing FAA fixative. The slices were prepared by paraffin sectioning method, observed, measured and micrographically photographed with an Olympus BX53 fluorescent microscope (Li et al., 2022a), and the thickness of leaf spongy tissue (μm), fenestrated tissue thickness (μm), leaf thickness (μm), main vein thickness (μm), root diameter (μm), stem diameter (μm), and stem epidermal cell thickness (μm) were measured and counted.

### 2.5 Cold-tolerant mutant screening and identification

#### 2.5.1 Low temperature stress treatment

The materials were identified mutant plants, seedlings aged 120d, and un-mutagenized wild-type plants of the same age were used as controls, and three pots of uniform growth each were selected for the cold stress experiment. Pre-treatment first: the mutant and control plants were pre-cultured in a light incubator (light intensity 3000 lx, light time 14h, day/night temperature 25/20°C, relative humidity 65%) for 3 d. Then the temperature was gradually lowered by 5°C for every 5 h interval (Zheng et al., 2022), and after 24h, the temperature was reduced to 0°C, at which point the low-temperature stress treatment was carried out: the design was 0°C with full dark period treatment. Ambient temperature of 25°C and a photoperiod of 12/12h were used as controls. After 48h of incubation, the plants were placed at room temperature for 24h to recover for morphological observation, and the leaves of mutant and control plants were taken simultaneously by punching method, snap frozen in liquid nitrogen and stored in -80°C refrigerator for physiological index determination.

#### 2.5.2 Preliminary identification of cold hardiness of mutant appearance and morphological index after low temperature treatment

During the low-temperature stress treatment, the morphological characteristics of *Plumbago indica* L. mutants and wild-type plants were observed, including leaf shape, leaf color, degree of leaf chlorosis, and curling or not. After the low-temperature treatment, the morphological injury indexes were observed after 24h of recovery at room temperature, and the preliminary identification of cold tolerance was carried out.

#### 2.5.3 Identification of physiological and biochemical indicators of cold resistance in mutants after low temperature treatment

Seven time periods, 0, 3, 8, 16, 24, 36, and 48 h, were selected during the low temperature stress treatment for the determination of chlorophyll fluorescence kinetic parameters (Li et al., 2022b). The fluorescence parameters such as Fo, Fm, Fm/Fo, Fv/Fm were measured by Handy PEA plant efficiency meter after 30 min of dark acclimation by taking the vigorously growing leaves without pests and diseases in the upper middle part of the plant, avoiding the leaf veins as much as possible and making the leaves be evenly clamped in the leaf clips when measuring. In addition, the leaves of the mutant and control plants were taken after 48 h of low temperature treatment and 24 h of recovery, and the two materials at room temperature were used as controls (Peng et al., 2018). The MDA content was determined using the thiobarbituric acid (TBA) method (Janero, 1990; Chen et al., 2020a) SOD, CAT and POD activities, and soluble sugar content and proline (Pro) content were determined using kits (Li et al., 2022b).

#### 2.5.4 Observation on field morphological indicators of cold resistant mutants

The consistent growth of *Plumbago indica* L. control and four mutant strains were selected for morphological observation. Three pots of each strain were selected and each pot was a biological replicate. Measurement method reference Shen et al. (2020).

Plant height: The vertical distance (cm) from the collar to the top of the plant was measured under natural conditions (no support with flower racks) (estimation: 0.01 cm).

Crown diameter: Based on the vertical projection as a theory, the length of longest axis and its horizontal and vertical axis are recorded and the average value (estimation: 0.01cm) was taken.

Number of inflorescences and inflorescence length: The number of inflorescences per pot was recorded, while the distance from the bottom of a single inflorescence to the base of the top flower was randomly measured in each pot and recorded as inflorescence length (estimation: 0.01cm).

Flower length: The vertical distance from the base of the sepals of three fully open flowers to the top of a single flower was measured randomly per pot (mm).

Flower diameter: The maximum diameter of the flower (mm) was measured for three fully open flowers randomly per pot.

#### 2.5.5 Data processing

CTR = fence tissue thickness/leaf thickness × 100%

SR=sponge tissue thickness/leaf thickness×100%

MDA concentration: C(μmol/L) =6.45(A_532_-A_600_)-0.56A_450_

MDA content ratio in fresh weight: MDA = C (μmol/L) × volume of extraction solution (L)/fresh weight of leaf tissue (g)

CAT (U/g fresh weight) = 764.5 × ΔA/leaf tissue fresh weight (g)

In the formula: ΔA: the difference between the OD value before and after the reaction.

POD (U/g fresh weight) = 9800 × ΔA/leaf tissue fresh weight (g)

In the formula: ΔA: the difference between the OD values before and after the reaction.

Soluble sugar content (%) = (C × V/a × n) (W×1000)

In the formula: C: standard equation for the amount of sugar (μg); a: volume of aspirated sample liquid (mL); V: volume of extraction solution (mL); n: dilution times; W: weight of tissue (g).

Proline (μg/g FW or DW) = C × V/a × W

In the formula: C: standard equation for pro volume (μg); V: volume of extraction solution (mL); a: volume of aspirated sample liquid (mL); W: weight of tissue (g)

Data from this study were correlated using SPSS 26.0 software and analyzed for significance (*P <*0.05), with Excel 2013 for data statistics and Origin9 for graphs. All data were subjected to three biological replicates, and the analysis is expressed as s ± $\overset{-}{\text{x}}$ (standard deviation).

The band loci were generated according to ISSR-PCR amplification, and the band polymorphisms were counted. In the same loci, the presence of the strip loci was recorded as “1” and the absence was recorded as “0” to construct a binary data matrix, while the genetic similarity coefficient of Dice was calculated using the biological software NTSYS-pc2.10, and the unweighted group average method (UPGMA) was used to cluster the ISSR-PCR amplified bands for genetic similarity analysis and used construct a dendrogram by the Mega.

## 3 Results

### 3.1 ISSR primer screening and polymorphism analysis

**Figure 3:**
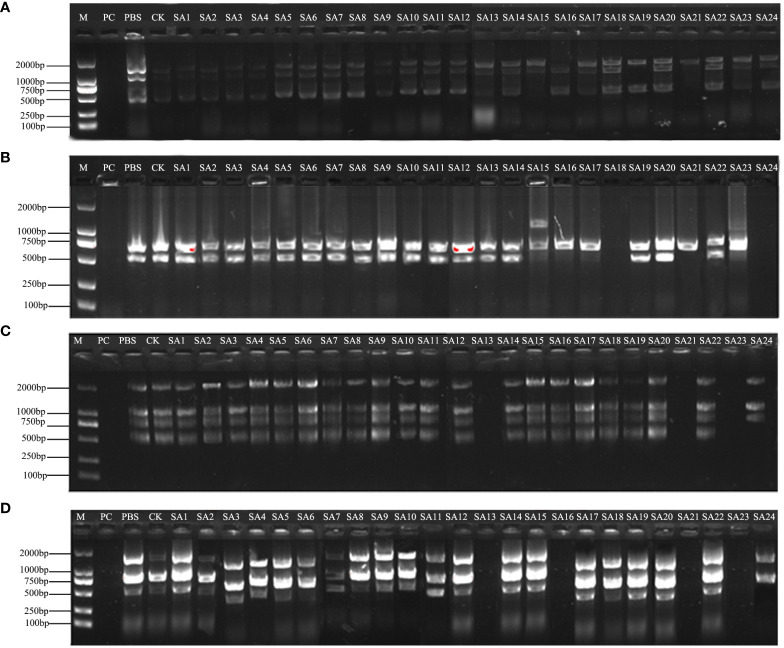
Amplification profile of ISSR partial primers. PC is the positive control, PBS is the control with phosphate buffer as the solution, and CK is the wild-type control. **(A–D)** are the amplification profiles of primers UBC807, UBC818, UBC866, UBC874, respectively.

**Figure 4:**
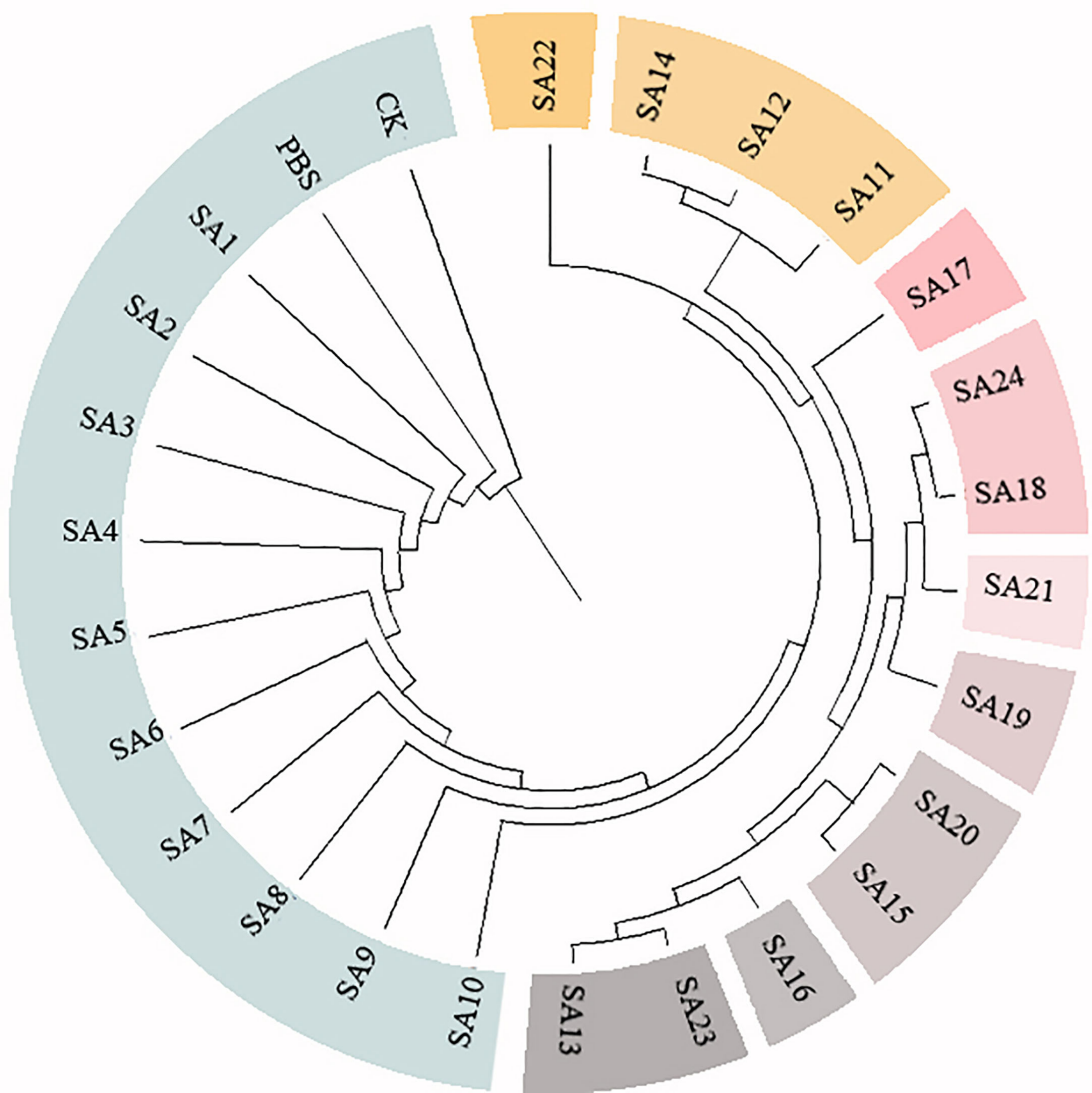
Cluster analysis of 24 suspected mutants of *Plumbago indica* L. and wild materials. 24 putative mutants (SA1 to SA24) derived from simple intersequence repeats (ISSR), 2 controls (CK, PBS); horizontal coordinates are genetic distances.

PCR amplification of wild-type DNA was performed with 100 UBC (University of British Columbia) ISSR universal primers, and the amplification products were detected by agarose gel electrophoresis. 9 primers, UBC807, UBC811, UBC818, UBC826, UBC844, UBC866, UBC874, UBC888, and UBC891, showed the best amplification results (**Table 1**), and it was finally determined that these 9 ISSR primers were used for PCR amplification of the suspected mutant of *Plumbago indica* L. ddH_2_O control under the same conditions to exclude false positive results. The amplification products range from 500bp to 2000bp (**Figures 3**, **4**).

**Table 1:** ISSR primer screening results and primer sequences.

Primers	Primer sequences
UBC807	AGA GAG AGA GAG AGA GT
UBC811	GAG AGA GAG AGA GAG AC
UBC818	CAC ACA CAC ACA CAC AG
UBC826	ACA CAC ACA CAC ACA CC
UBC844	CTC TCT CTC TCT CTC TRC
UBC866	CTC CTC CTC CTC CTC CTC
UBC874	CCC TCC CTC CCT CCC T
UBC888	BDB CAC ACA CAC ACA CA
UBC891	HVH TGT GTG TGT GTG T

The screened nine ISSR universal primers (**Table 1**) were used to PCR amplify the DNA of 24 *Plumbago indica* L. suspected mutant materials, and a total of 32 bands were amplified, of which 28 were polymorphic bands with a polymorphism ratio of 87.5%, indicating that the test materials were rich in variation. Each primer could amplify 2-5 bands, with an average of 3.6 bands and an average of 3.1 polymorphic bands, among which UBC888 amplified the most bands and polymorphic bands, both of which were 5, as detailed in **Table 2**.

**Table 2:** Amplification polymorphism analysis of 24 suspected mutant strain materials by 9 primers of ISSR.

Primers	Number of amplified bands	Number of polymorphic bands	Polymorphism ratio (%)
UBC807	3	3	100.0
UBC811	4	2	50.0
UBC818	3	3	100.0
UBC826	4	4	100.0
UBC844	2	1	50.0
UBC866	4	4	100.0
UBC874	3	3	100.0
UBC888	5	5	100.0
UBC891	4	3	75.0
Total	32	28	87.5

The results of partial ISSR amplification of 26 (including 2 controls) materials with 9 primers are shown in **Figure 3**, and some of the 24 strains showed band increases and deletions. For example, under UBC818 amplification, material SA15 has one more band between 1000-2000 bp compared with the wild type, material SA16, SA17, SA21 and SA23 have one missing band between 500-750 bp, and material SA18 and SA24 have completely missing bands; under UBC866 amplification, material SA24 has one more band between 500-750 bp compared with the wild type. Under UBC866 amplification, material SA24 is missing one band between 500-750 bp compared with wild type, and SA13, SA21, and SA23 are completely missing. These examples indicate that these numbered materials are likely to be mutants.

### 3.2 Genetic similarity and clustering analysis

Based on the 28 polymorphic sites amplified by the 9 primers, the genetic similarity coefficients of the 24 suspected mutations and the 2 wild type materials were calculated and were shown in the **Supplementary Material**. Based on the ISSR genetic similarity coefficients, the cluster analysis of the 26 test materials constructed using UPGMA by the Mega unweighted arithmetic mean method was shown in **Figure 4**, which could be classified into 10 classes, among which SA1-SA10 had close genetic coefficients to CK and PBS and were the most closely related, followed by SA22, and SA11, SA12, and SA14. The remaining 9 materials had low genetic similarity coefficients and were more distantly related to CK.

In conclusion, after further molecular marker identification and cluster analysis of the 24 suspected mutants initially identified by morphology, 9 materials SA13, SA15, SA16, SA18, SA19, SA20, SA21, SA23, and SA24 could be tentatively judged as mutant strains of *Plumbago indica* L.

### 3.3 Analysis of plumbagin content in stem and leaves of mutants

**Figure 5:**
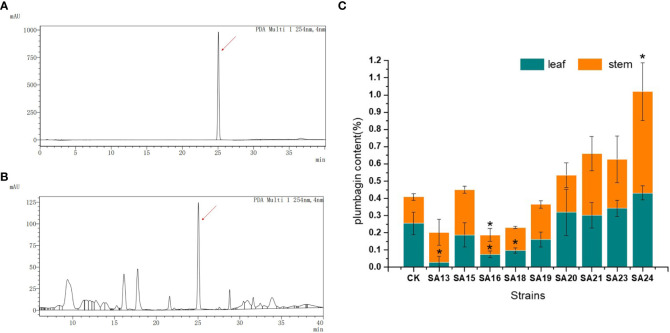
Plumbagin content of 9 mutants in stems and leaves. Note: **(A)** the peak plot of plumbagin standard product; **(B)** the peak plot of the plant sample (CK as an example); **(C)** The comparison chart of plumbagin content between plant samples; “*” means with significant differences between different mutants and the control group ( at P<0.05).

The contents of the medicinal component plumbagin in control and nine mutant strains were detected, as shown in **Figures 5A, B**. As with the standard solution, both stem and leaf extracts of control plants and mutant strains had signal peaks at about 25 min, indicating that the mutant stems and leaves contained plumbagin. The content of plumbagin in the leaves was calculated (**Figure 5C**), and it was found that except for SA13, SA16 and SA18, which were significantly lower than CK, the content of all plants was not significantly different compared with CK, indicating that mutagenesis affected the accumulation of plumbagin in the leaves of SA13, SA16 and SA18. In the stems, only SA24 had significantly higher plumbagin content than CK, 0.5991%, which was 3.84 times higher than the control, while SA16 was significantly lower than the control group. For the above-ground parts, the total plumbagin content was highest in SA24, followed by SA21. Therefore, SA15, SA19, SA20, SA21, SA23 and SA24 can be continuously observed as better mutants, provided that their medicinal value is not affected.

### 3.4 Characterization of the anatomical features of the nutrient organs of nine mutants of *Plumbago indica* L.

#### 3.4.1 Anatomical features of the root

**Figure 6:**
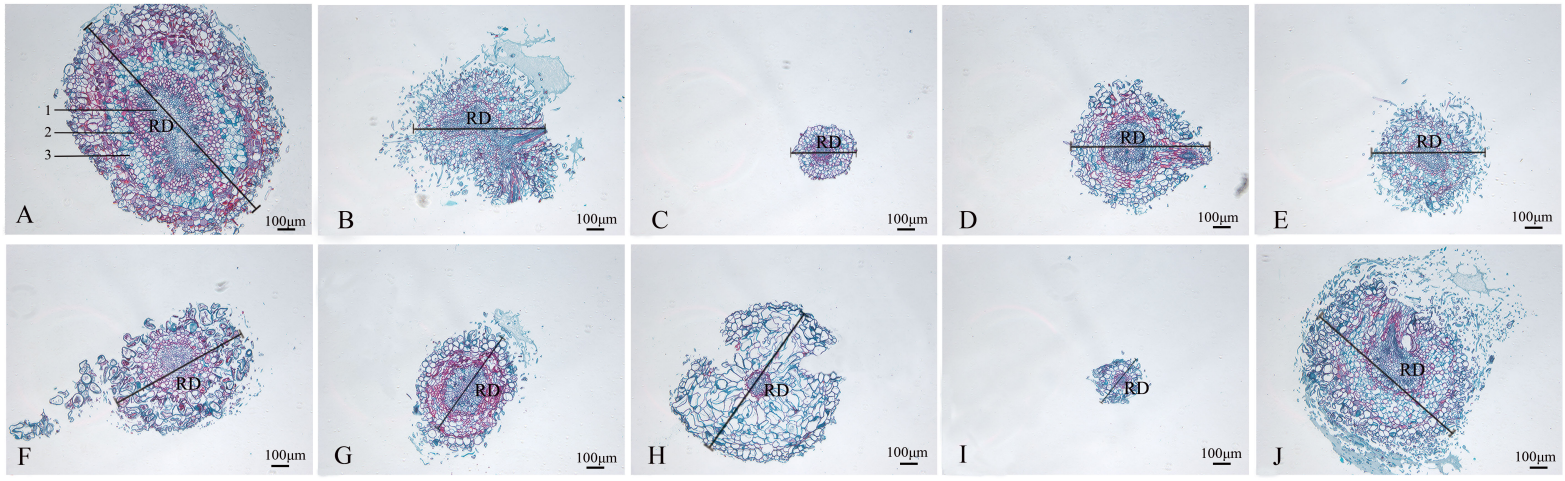
Comparison of the anatomical and structural characteristics of the roots of nine *Plumbago indica* L. mutants with the wild type: *Root cross-sections of CK, SA13, SA15, SA16, SA18, SA19, SA20, SA21, SA23, SA24 **(A–J)**; Root diameter (RD); In **(A)**, 1 is the xylem of the root, 2 is the cambium, and 3 is the phloem..

The wild strain’s roots of the nine mutants identified by ISSR molecular markers were separated, and their anatomical characteristics are displayed (**Figure 6**; **Table 3**). The xylem, vascular cambium, and phloem cells in the root cross-section of CK are all tightly packed and clearly bound. Compared with CK, mutants SA13, SA16, SA18, SA19, and SA24 (**Figures 6B, D–F, J**) had tightly arranged xylem cells and reduced the number of cambium cell layers in the vascular bundle, and SA13 root cross-sections were irregularly elliptical. SA15 and SA23 root cross-sectional areas were significantly reduced (**Figures 6C, I**; **Table 3**), and SA23 phloem cells were large and had a reduced number of cell layers. SA21 (**Figure 6H**) has a small xylem area, small and dense cells, and is closely surrounded by vascular cambium; the cells of the phloem are large, and this part accounts for the majority of the root cross-sectional area.

**Table 3:** Root diameter of nine mutants and wild-type material of *Plumbago indica* L.

Strain	Root diameter(μm)
CK	1337.86 ± 18.76^a^
SA13	811.66 ± 20.99^d^
SA15	317.08 ± 8.92^h^
SA16	656.14 ± 21.04^e^
SA18	507.31 ± 7.73^f^
SA19	435.41 ± 21.68^g^
SA20	506.28 ± 13.32^f^
SA21	926.53 ± 14.98^c^
SA23	228.89 ± 9.64^i^
SA24	961.40 ± 12.09^b^

The same letters are non-significantly different for different mutants and controls (at P<0.05).

Root cross-sections of CK, SA13, SA15, SA16, SA18, SA19, SA20, SA21, SA23, SA24(a-j); Root diameter(RD); In **Figure 3A**, 1 is the xylem of the root, 2 is the cambium, and 3 is the phloem.

#### 3.4.2 Anatomical features of the stem

**Figure 7:**
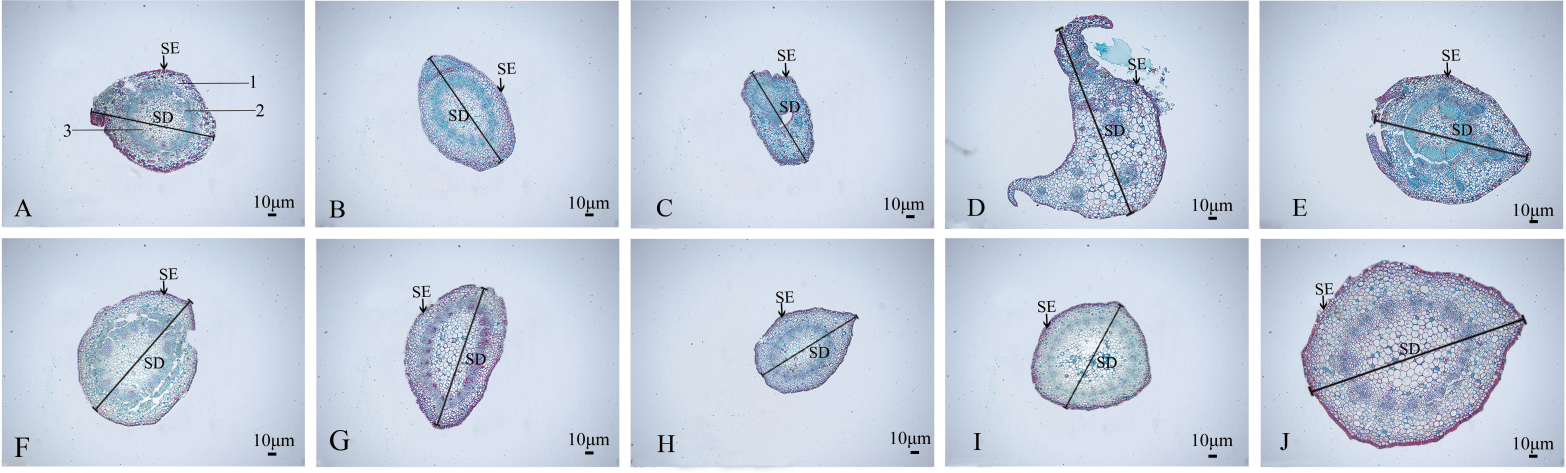
Comparison of the anatomical and structural characteristics of the stem of nine *Plumbago indica* L. mutants with the wild type: Stem cross-sections of CK, SA13, SA15, SA16, SA18, SA19, SA20, SA21, SA23, SA24 **(A–J)**; Stem epidermal cells(SE); Stem diameter(SD); **(A)** 1 is the xylem of the stem, 2 is the cambium, and 3 is the phloem.

The stem anatomical features of the nine mutant and wild-type materials are displayed (**Figure 7**; **Table 4**). Stem diameter and stem epidermal cell thickness showed significant differences (*P*<0.05) from CK, manifested as an increase (7 plants) or decrease (2 plants) in stem diameter; among them, SA13, SA16, SA20, and SA24 (**Figures 7B, D, G, J**) showed significant differences (*P*<0.05) from wild type in stem epidermal cell thickness, and SA24 was the most significant in both stem diameter and stem epidermal cell thickness, with 1.19-fold and 1.15-fold increase respectively. The cross-sectional shape did not change significantly in the other eight mutant materials compared with CK, except for SA16 (**Figure 7**). On the cell arrangement characteristics, the xylem, cambium, and phloem of SA15 showed no ring arrangement, and the collenchyma on one side was obviously thickened, and the epidermal cells formed in the middle divided the transverse section of the stem into two parts (**Figure 7C**). SA16 has a “lunar” shaped stem cross-section, with xylem, cambium, and phloem scattered in the middle expansion area and abnormal distribution of cellular tissue structure (**Figure 7D**). The collenchyma on one side of SA18 was thickened and irregularly distributed on the thickened side in addition to the circularly arranged xylem, cambium, and phloem. The rest of the mutant strains showed no significant abnormalities compared with the CK, and only differences in the degree of compactness of collenchyma and cortical cell arrangement were observed (**Figure 7E**).

**Table 4:** Parameters related to the stem anatomical features of nine putatively mutants and wild type of *Plumbago indica* L.

Strain	Stem diameter(μm)	Stem epidermal cell thickness(μm)
CK	239.38 ± 1.81^g^	3.33 ± 0.41^e^
SA13	333.54 ± 4.31^c^	4.91 ± 0.48^bc^
SA15	217.56 ± 6.86^h^	3.43 ± 0.15^de^
SA16	221.31 ± 2.99^h^	5.34 ± 1.11^b^
SA18	321.50 ± 9.06^d^	3.81 ± 0.59^de^
SA19	328.86 ± 2.11^cd^	3.63 ± 0.59^de^
SA20	350.14 ± 6.52^b^	4.21 ± 0.50^cd^
SA21	258.68 ± 7.38^f^	3.25 ± 0.40^e^
SA23	279.66 ± 6.16^e^	3.64 ± 0.36^de^
SA24	525.28 ± 8.41^a^	7.18 ± 0.78^a^

The same letters are non-significantly different for different mutants and controls (at P<0.05).

#### 3.4.3 Anatomical features of the leaf

**Figure 8:**
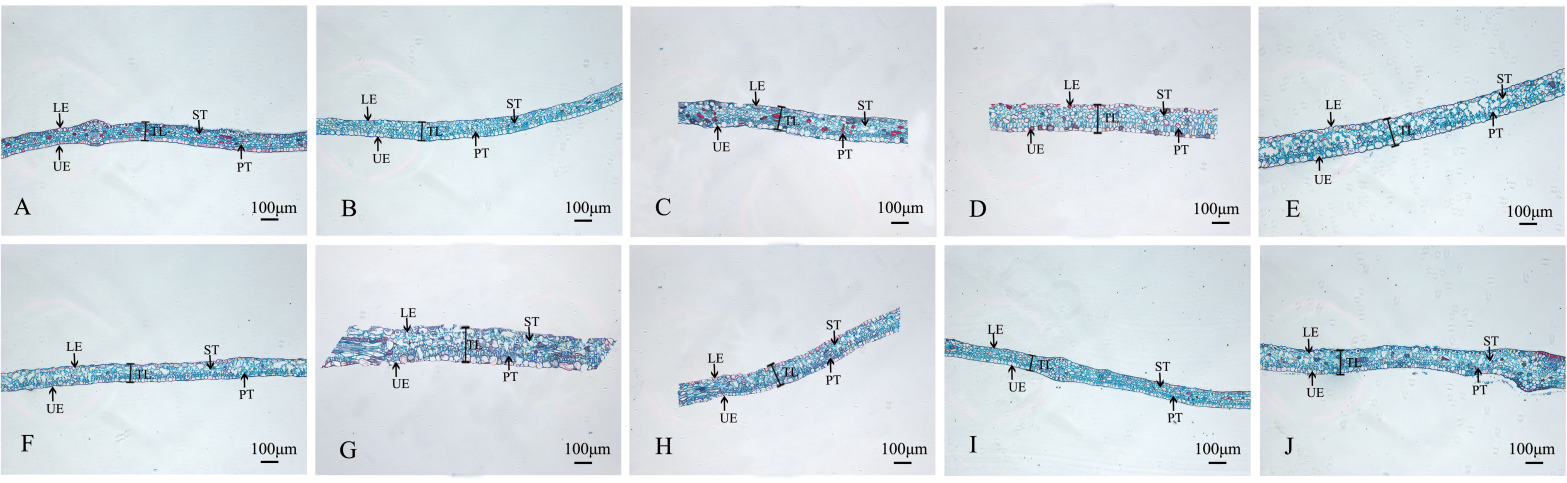
Comparison of the mesophyll cross-sections of nine *Plumbago indica* L. mutants with the wild type Mesophyll cross-sections of CK, SA13, SA15, SA16, SA18, SA19, SA20, SA21, SA23, SA24 **(A–J)**; Upper Epidermal(UR); Lower Epidermal (LE); Leaf Thickness(LT); Spongy Tissue(ST); Palisade Tissue(PT).

The structures of mesophyll and leaf main vein cross-sections of nine mutant and wild-type leaves are shown (**Figure 8**; **Table 5**). The mutant plants exhibited changes in leaf thickness and main vein cross-sectional size; the results of statistical analysis of the relevant parameters are shown in **Table 5**.

**Table 5:** Parameters related to the leaf anatomical features of nine mutants and wild type of *Plumbago indica* L.

Strain	Leaf thickness (μm)	Main vein thickness (μm)	Upper epidermal cell thickness (μm)	Lower epidermal cell thickness (μm)	Spongy tissue thickness (μm)	Palisade tissue thickness (μm)	CTR (%)	SR (%)
CK	101.44 ± 1.13^f^	394.62 ± 13.14^h^	18.26 ± 1.15^ab^	21.88 ± 14^cde^	41.13 ± 3.36^f^	25.01 ± 2.43^cd^	24.65 ± 2.27^ab^	40.56 ± 3.44^d^
SA13	102.25 ± 0.56^f^	544.50 ± 11.47^e^	17.39 ± 1.17^ab^	20.83 ± 1.36^de^	40.30 ± 4.91^f^	27.72 ± 3.21^c^	27.11 ± 3.15^a^	39.42 ± 4.86^d^
SA15	145.26 ± 13.37^d^	980.33 ± 23.04^a^	18.71 ± 0.53^ab^	26.98 ± 2.73^ab^	61.01 ± 3.82^d^	28.38 ± 4.17^c^	19.64 ± 3.21^c^	42.19 ± 3.94^cd^
SA16	156.72 ± 5.31^c^	380.50 ± 0.85^h^	19.73 ± 0.55^ab^	28.91 ± 1.71^a^	68.90 ± 3.21^c^	38.29 ± 6.29^a^	24.24 ± 4.62^ab^	43.99 ± 2.17^cd^
SA18	169.82 ± 9.63^b^	729.35 ± 13.68^c^	21.80 ± 2.92^a^	28.75 ± 3.15^a^	77.66 ± 6.21^ab^	33.82 ± 5.14^b^	19.85 ± 2.58^c^	45.83 ± 4.36^c^
SA19	103.95 ± 3.95^f^	448.97 ± 7.22^g^	19.55 ± 0.99^ab^	26.23 ± 1.74^ab^	54.17 ± 5.25^e^	27.62 ± 1.94^c^	26.62 ± 2.42^a^	52.16 ± 2.57^b^
SA20	185.63 ± 5.02^a^	950.37 ± 15.42^b^	20.08 ± 2.07^ab^	23.13 ± 1.90^bcd^	81.07 ± 4.68^a^	23.97 ± 2.10^cd^	12.93 ± 1.14^d^	43.68 ± 2.37^cd^
SA21	135.09 ± 2.98^de^	491.13 ± 4.46^f^	18.01 ± 2.39^ab^	25.31 ± 3.08^abc^	74.92 ± 3.69^b^	21.62 ± 3.30^d^	16.01 ± 2.49^d^	55.46 ± 3.02^ab^
SA23	98.46 ± 6.66^f^	561.88 ± 3.99^e^	16.11 ± 0.93^b^	18.59 ± 2.27^e^	41.57 ± 2.66^f^	24.53 ± 0.96^cd^	27.93 ± 4.67^a^	47.13 ± 3.75^c^
SA24	127.60 ± 1.36^e^	667.99 ± 13.81^d^	21.61 ± 2.67^a^	20.98 ± 1.21^de^	73.48 ± 5.19^bc^	27.53 ± 5.77^c^	21.62 ± 0.56^bc^	57.70 ± 4.03^a^

The same letters are non-significantly different for different mutants and controls (at P<0.05).

In terms of leaf thickness, only SA13, SA19 and SA23 were not significantly different from the wild type, while the rest were significantly thicker than the wild type; leaf main vein thickness was significantly greater than the wild type except for SA16. The largest of them was SA15, reaching 980.333 μm, which was 1.48 times more than the wild type. As can be seen, most of the mutant strains exhibited plump leaves with prominent veins.

The microstructure also showed some differences. The lower epidermis was thicker than the upper epidermis in general, and the thickness of the upper epidermis cells ranged from 16.1063-21.8013 μm, which was not significantly different from CK. The thickness of the lower epidermal cells ranged from 18.59-28.91 μm, with only SA16, SA18 and SA19 mutant strains being thicker than the wild type that showed significant differences. Compared with the CK spongy tissue thickness, seven of the nine materials showed thickening, SA20 thickness was the largest, and the cells were more loosely arranged at 81.0693 μm, which was a 0.97-fold increase; the palisade tissue thickness was no difference among the seven materials.

Analysis of cell tense ratio (CTR) and spongy ratio (SR) of the nine mutant materials and the wild type showed that only SA16 and CK had no significant difference in CTR and SR. In terms of CTR, SA23 had the largest value of 27.93%, followed by SA13, SA19, SA16, and SA24 in descending order, all of which were not significantly different from the wild type. In terms of SR, it was found that SA24 had the largest value, followed by SA21, SA19, SA23, and SA18 in descending order, all of which were larger and significantly different than CK.

In summary, the nine mutant materials differed significantly from the wild type in the anatomical features of the leaf.

**Figure 9:**
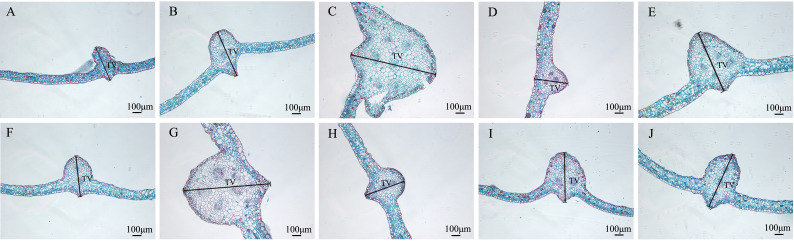
Comparison of the main leaf vein cross-sections of nine *Plumbago indica* L. mutants with the wild type. * Main leaf vein cross-sections of CK, SA13, SA15, SA16, SA18, SA19, SA20, SA21, SA23, SA24**(A–J)**; Thickness of main vein(TV).

Combining the anatomical features of the mutant material in the root, stem, and leaf nutrient organs (**Figures 8**, **9**), it can be seen that, compared with the wild type, the thickness of the root of the mutant strain is smaller than that of the wild strain, and there are significant differences; the stem diameter is generally thicker, with SA24 being the most prominent in terms of stem diameter and stem epidermal cell thickness. The leaves mostly changed morphologically, as shown by an increase in leaf thickness, and the microstructure showed the same trend. In summary, the nine mutant materials differed significantly from the wild-type plants in the three nutritional organs of roots, stems, and leaves, both in macroscopic and microscopic anatomical features, and this result confirmed the reliability of the ISSR molecular marker identification results. The nine *Plumbago indica* L. materials, SA13, SA15, SA16, SA18, SA19, SA20, SA21, SA23 and SA24, were further identified as mutants.

### 3.5 Changes in the morphological characteristics of *Plumbago indica* L. mutants under low-temperature stress

**Figure 10:**
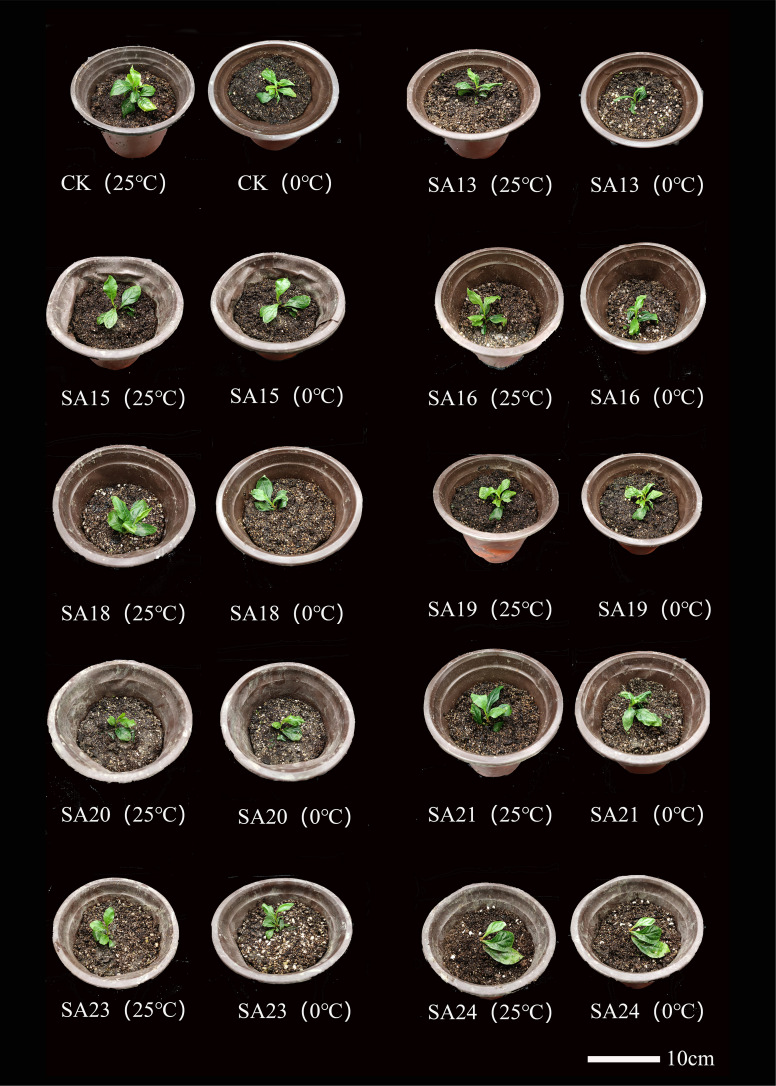
Comparison of the morphology of mutant material and wild type of *Plumbago indica* L. under low-temperature stress.

The morphological changes of the mutants and wild type after low-temperature stress are shown (**Figure 10**). Compared with CK, all nine mutant and wild-type plants were damaged to different degrees, showing leaf crinkling and wilting, yellowing of leaves, and stem collapse, with SA13 and SA18 being more severely damaged than the wild type. After 24 h of rewarming, the mutants SA15, SA19, SA23 and SA24 were found to have only slightly crinkled leaves and slightly yellowed leaves, and the damage was weaker than that of wild-type plants. We tentatively judged that these four mutants could be the focus of observation.

### 3.6 Changes in the physiological and biochemical characteristics of *Plumbago indica* L. mutants under low-temperature stress

#### 3.6.1 Chlorophyll fluorescence kinetic parameters of the mutant

**Figure 11:**
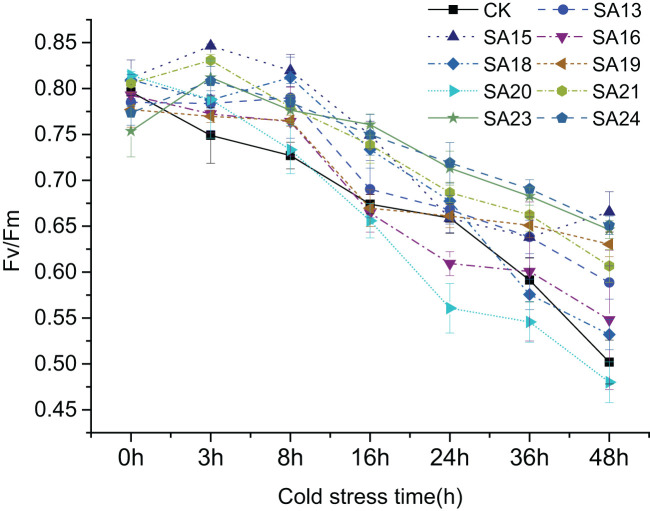
Variation of Fv/Fm in leaves of *Plumbago indica* L. mutants and wild material under low-temperature stress.

It shows the changes of Fv/Fm in the leaves of nine mutant materials and wild-type plants under low-temperature stress(**Figure 11**), and it can be seen that after 48 h of low-temperature treatment, there was an overall decreasing trend, from 0.75-0.81 before treatment to between 0.48-0.66. Except for SA20 which Fv/Fm value decreased by 50.42%, which was greater than CK (0.40, 46.41%), the other eight mutant materials decreased by less than CK, between 18.90% and 42.34% indicated that the mutant plant as a whole had a stronger ability to cope with short-term low-temperature stress than CK, among which SA23 decreased the least compared with CK, 18.90%, and the damage of photosynthesis was the least. This was followed by SA24 (20.49%), SA15 (22.16%) and SA19 (24.29%). It can be seen that the mutant materials generally have better cold tolerance than the wild type, among which SA15, SA19, SA23 and SA24 have higher light energy conversion efficiency and better ability to cope with low-temperature stress.

#### 3.6.2 Malondialdehyde content of *Plumbago indica* L. mutants under low-temperature stress

**Figure 12:**
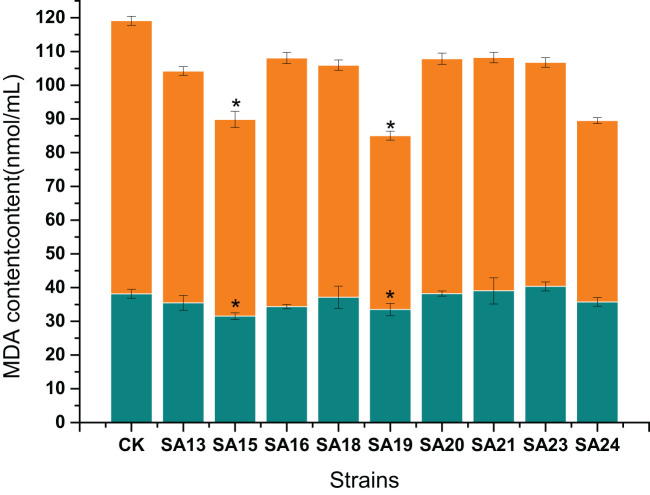
Effect of low-temperature stress on the MDA content of mutant material of *Plumbago indica* L.. “*” means with significant differences between different mutants and the control group (at *P*<0.05). The green part is the MDA content of each strain before treatment, the total column length is the MDA content of each strain after treatment, and the orange part is the relative MDA content.

The changes in MDA content are shown (**Figure 12**). Before the treatment without low-temperature stress, the MDA content of both the treatment group and CK was low. After 48 h of low-temperature stress treatment, the MDA content in the leaves of all materials increased rapidly, the relative MDA content and the rise in CK were 42.82 mmol/L and 112.21%, respectively. The mutant materials increased between 50.00% and 114.00%, except for SA16, the rest eight mutant materials increased less than the wild strain and showed better resistance than the wild type, among which, SA24 MDA relative content increased the least, 17.99%, indicating the lowest degree of cell membrane injury and the strongest resistance to low-temperature stress, followed by SA15, SA19, and SA23, with relative MDA content increased by 26.80 mmol/mL, 18.06 mmol/mL, and 26.07 mmol/mL, respectively.

#### 3.6.3 SOD, CAT, and POD activities of *Plumbago indica* L. mutants under low-temperature stress

**Figure 13:**
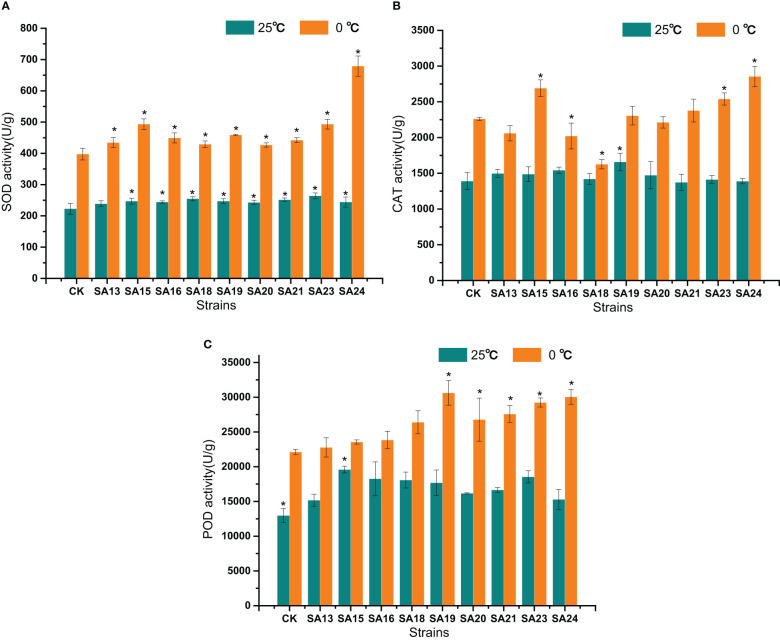
Effects of low-temperature stress on SOD **(A)**, CAT **(B)**, and POD **(C)** activities of mutant material of *Plumbago indica* L.. “*” means with significant differences between different mutants and the control group (at *P*<0.05).

The changes of SOD, CAT, and POD activities after low temperature stress in the nine mutant materials and the control are shown in **Figure 13**. Before low temperature stress treatment, all mutant strains except SA13 had significantly higher SOD activity than CK (*P*<0.05) (**Figure 13A**); only SA19 had significantly higher CAT activity than CK(**Figure 13B**); only SA15 had significantly higher POD activity than CK (**Figure 13C**). After 48 h of low temperature stress treatment, SOD activity in the leaves of all materials increased rapidly, and all mutant strains were higher than that of CK, among which the highest SOD activity was SA24, with 679.04 U/g, was 1.09 times higher than that of CK after stress, followed by SA15 (493.25 U/g, 0.62 times), SA23 (493.23 U/g, 0.58 times), and SA19 (458.74 U/g, 0.53 times); CAT activity showed that SA15, SA23, and SA24 were significantly higher than that of the control (*P*<0.05), with 429.71 U/g, 278.985 U/g and 593.316, respectively. while the CAT enzyme activities of SA16 and SA18 were significantly lower than those of the control (*P*<0.05); in the performance of POD activities, all four mutant strains of SA19-SA24 were significantly higher than that of CK (*P*<0.05), and SA19 was the highest at 30627.76 U/g, followed by SA24 at 30052.09 U/g.

#### 3.6.4 The soluble sugar content of *Plumbago indica* L. mutants under low-temperature stress

**Figure 14:**
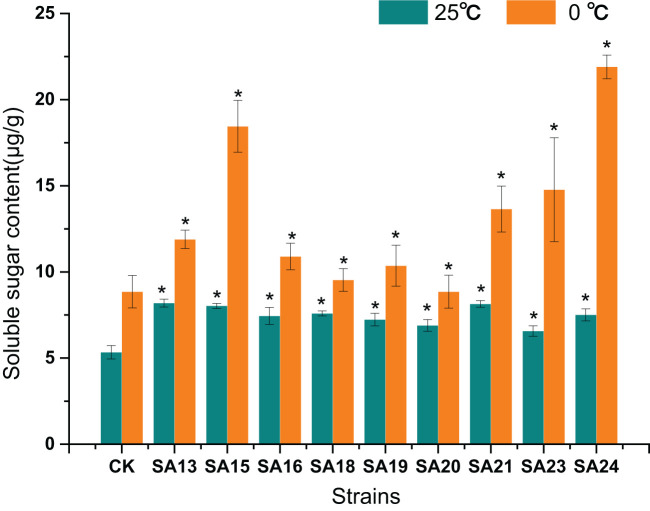
Effect of low-temperature stress on the soluble sugar content of mutant material of *Plumbago indica* L.. “*” means with significant differences between different mutants and the control group (at *P*<0.05).

None of the mutant materials had high soluble sugar content before low-temperature stress, but all were significantly higher than CK (*P*<0.05), ranging from 5.33-8.14 μg/g. After 48 h of low-temperature stress, the contents of both mutants and CK increased significantly (*P*<0.05), and the contents of mutant materials rose to between 8.85-21.9 μg/g, with SA24 showing the highest increment of 14.39 μg/g, a 1.92-fold increase compared to CK, followed by SA15 (10.42 μg/g, 1.30-fold), SA23 (8.21 μg/g, 1.25-fold), and SA21 (5.51 μg/g, 0.68-fold), indicating that they were stronger than CK in mobilizing osmoregulatory substances to resist low-temperature stress, which laterally reflected that their cold tolerance was also stronger than CK (**Figure 14**).

#### 3.6.5 Proline content of *Plumbago indica* L. mutants under low-temperature stress

**Figure 15:**
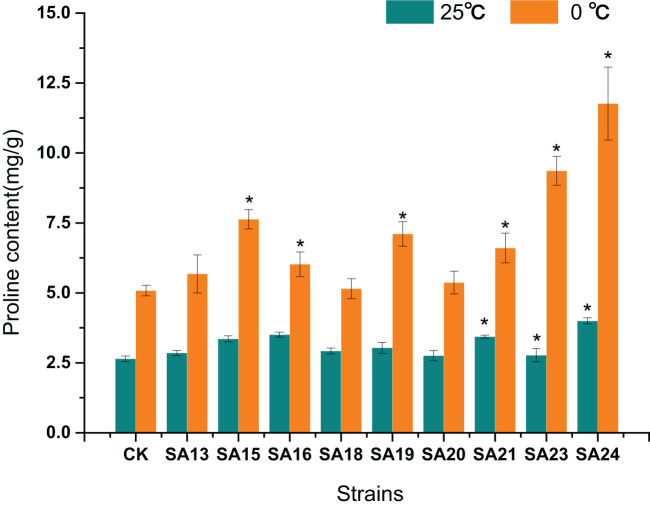
Effect of low-temperature stress on the proline content of mutant material of *Plumbago indica* L.. ”*“ means with significant differences between different mutants and the control group (at *P*<0.05).

After low-temperature stress treatment, the Pro content in CK increased from 2.64 mg/g to 5.08 mg/g, and compared with CK, the Pro content of all eight mutant materials increased significantly, except for SA13, SA16, and SA20, the increase was significantly greater (*P*<0.05). SA24 had the highest Pro content of 11.77 mg/g, a2.74-fold increase compared to CK, followed one is SA23 (9.36 mg/g, 2.70-fold), SA15 (7.63 mg/g, 1.75-fold), and SA19 (7.11 mg/g, 1.67-fold). It reflected that these four mutants had a higher ability to withstand cold stress than CK from the side (**Figure 15**).

In conclusion, by analyzing the changes in chlorophyll fluorescence parameters, MDA, soluble sugar, and proline contents between the mutant and wild plant CK before and after the onset of low-temperature stress, it was evident that the two types of materials differed to different degrees in their cold tolerance. Compared with CK, some mutant materials showed weaker cold tolerance and some showed stronger cold tolerance. Taken together, the four mutant materials SA15, SA19, SA23, and SA24 showed higher light energy conversion efficiency, less accumulation of MDA content, have the capable of actively mobilizing the activity of antioxidant enzymes. and they also have more accumulation of soluble sugar and proline content in response to low-temperature stress, and did not affect or even exceed the accumulation of plumbagin in stems and leaves in control. They showed some cold tolerance at the level of physiological and biochemical characteristics, which was more consistent with the morphological observation of low-temperature injury performance. Therefore, these four mutants can be used as backup materials for the screening of cold-tolerant *Plumbago indica* L. varieties for subsequent field observations.

#### 3.6.6 Observation on morphological indicators in the field of cold-resistant mutants

**Figure 16:**
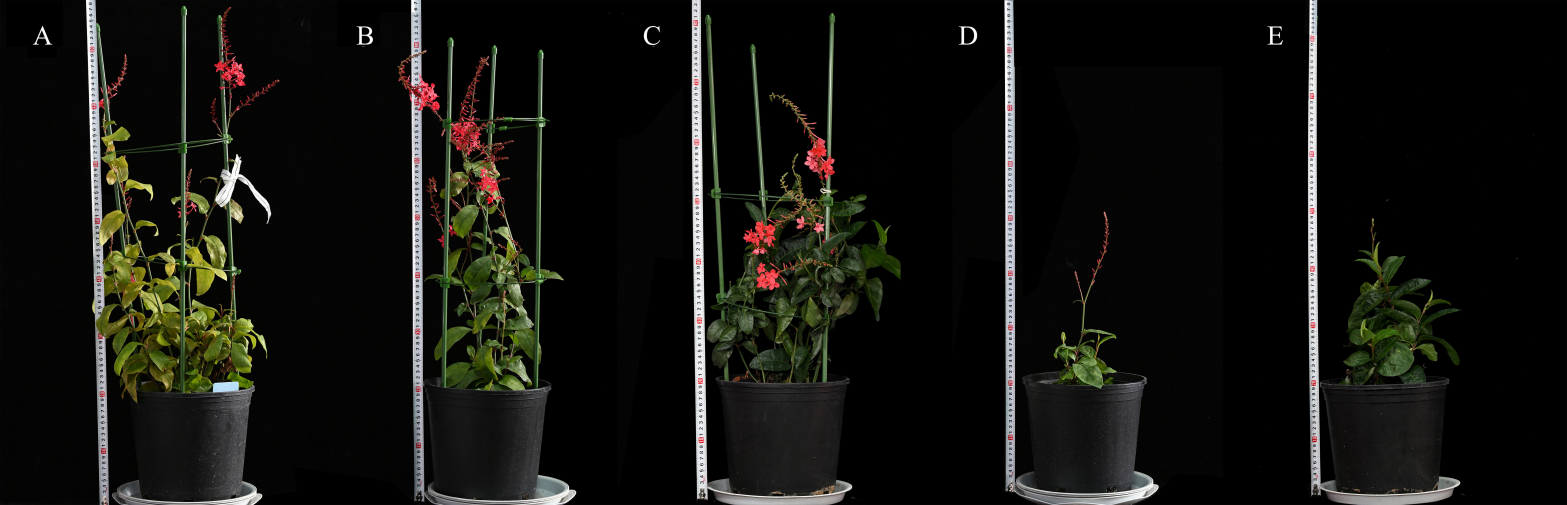
Comparison of the growth of the four *Plumbago indica* L. mutant materials at the end of November 2022 (12°C-18°C) **(A–E)** in the figure are CK, SA23, SA15, SA19, and SA24, in that order.

Agronomic ornamental traits were observed in the end of November of the next year (temperature 12°C-18°C) for the cold-tolerant mutants grown in soil culture for one year. As shown in **Table 6** and **Figure 16**, the plant height of SA19 and SA24 was significantly lower than that of CK (*P*<0.05), which showed a dwarf and compact plant size; the crown diameter of SA19, SA23 and SA24 was significantly smaller than that of CK (*P*<0.05), and only SA15 retained the original crown diameter but the inflorescence length was significantly lower than that of control and more compact compared with control; SA19 and SA24 mainly showed lagging inflorescence growth. In **Figure 16**, the leaves of control gradually yellowed and wilted, and the flowers started to wither gradually; the leaves of the cold-tolerant mutant became green to dark green, and SA15 and SA23 were in full flowering stage, while SA19 and SA24 were under reproductive growth. Therefore, the cold-resistant mutant mainly showed the characteristics of delayed flowering and delayed senescence, and had a longer ornamental period.

**Table 6:** Statistics of morphological indicators of cold-tolerant mutants at the end of November.

Strain	Plant height(cm)	Crown diameter(cm)	Inflorescence number	Inflorescence length(cm)	Flower diameter(mm)	Flower length(mm)
CK	48.60 ± 1.55^a^	50.80 ± 2.23^a^	10.33 ± 2.52^a^	21.47 ± 5.86^a^	20.45 ± 1.02^a^	22.35 ± 3.33^a^
SA15	45.73 ± 1.31^ab^	51.80 ± 2.93^a^	13.67 ± 2.52^a^	14.59 ± 3.12^b^	19.98 ± 1.79^a^	24.20 ± 3.49^a^
SA19	33.83 ± 3.07^c^	19.80 ± 1.87^d^	3.67 ± 1.53^b^	–	–	–
SA23	43.47 ± 1.30^b^	43.63 ± 1.40^b^	11.33 ± 1.53^a^	23.21 ± 3.99^a^	21.25 ± 3.49^a^	23.80 ± 4.36^a^
SA24	35.90 ± 1.95^c^	28.13 ± 1.81^c^	1.00 ± 1.00^b^	–	–	–

”-“ means with the data is not available, and the same letters are non-significantly different for different mutants and controls. (at P<0.05).

## 4 Discussion

### 4.1 NaN_3_ mutagenesis is an effective method to obtain mutants of *Plumbago indica* L.

The genotoxicity of NaN_3_ has been extensively studied, and its produced metabolite β-azidoalanine fraction [N^3^-CH^2^-CH(NH^2^)-COOH] plays a mutagenic role, inducing chromosomal mutations at a lower rate than other chemical mutagens (Gomez et al., 2019). Thus NaN_3_ is characterized by random regions causing a high frequency of mutations and a low frequency of chromosomal abnormalities (Turkoglu et al., 2022). In this study, plants treated with NaN_3_ and low-temperature stress in the field mainly showed dwarf plants, slightly curled leaves and slightly yellowed leaves, consistent with the mutagenic characteristics of NaN_3_. The concentration of mutagen affects tissue survival and mutagenic capacity, and using the optimal concentration of mutagen is necessary to achieve successful mutagenesis by balancing reasonable survival rates and finding the desired mutant sample (Wannajindaporn et al., 2016). The LD_50_ concentration that results in 50% mortality is usually considered to be the better equilibrium point. The material for this experiment was derived from the optimal protocol 0.5 mmol/L, treated for 1h, at which time LD50 = 53.33%, closest to 50% mortality, and mutagenesis efficiency of 5% by morphological and anatomical features, mutants confirmed by the combination of ISSR molecular markers, corroborates the feasibility of the previous conclusions on the equilibrium point. Therefore, NaN_3_ was further demonstrated to be a better mutagen for mutant induction in plants (e.g. *Plumbago indica* L.) due to its low damage to genes and high mutagenic efficiency.

### 4.2 ISSR molecular markers can be an effective method for screening mutant strains of *Plumbago indica* L. at the seedling stage

ISSR amplification techniques are sensitive enough to distinguish closely related individuals and assess the genetic diversity of germplasm and an effective means of assessing mutants (Velu et al., 2008). ISSR and genetic distance analysis allow better determination of variations originating from the same genotype (Ziarovska et al., 2013). In this study, the disappearance of normal bands and the appearance of new bands due to the effect of NaN_3_ mutagen were found under the characterization of ISSR technique using NaN_3_ *in vitro* mutagenesis of *Plumbago indica* L. New bands may be associated with mutations, while loss of bands may be associated with DNA damage, DNA methylation and chromosomal damage (Turkoglu et al., 2022). In the dendrogram and genetic data analysis, it was demonstrated that the mutant strains SA19, SA16, SA15, SA20, SA18, SA24, SA21, SA13, and SA23 had sequentially increased genetic distances compared to the parent and that the mutual strains showed different physiological, biochemical, and anatomical characteristics, which also indicated the uniqueness of each mutant strain (Ziarovska et al., 2013). ISSR marker clustering analysis is associated with differences in phenotypic traits (Wu et al., 2020), and this identification technique can help distinguish between morphologically distinct plants; it can also provide a solution for early seedling targeted breeding to screen for mutants, as the undirected nature of mutagenesis leads breeders to overlook invisible resistance indicators. Sulu et al. (2020) also used this approach to demonstrate induced mutations and genetic differentiation of citrus and lemon mutant genotypes as an effective tool for determining plant genotypes. In this study, the initial screening of mutants by clever use of ISSR molecular markers was more advantageous than morphological markers, and the mutant strains initially identified by this method were corroborated by subsequent anatomical evidence, indicating the reliability of ISSR molecular marker identification. In addition, using this marker technology, that solves the hidden variation of non-appearance changes that are difficult to screen by *in vitro* mutagenesis, and can quickly locate the plant body at the seedling stage, which facilitates later narrowing down the observation and testing population and targeted screening of target plants. Therefore, we believe that ISSR molecular markers can be used as a reliable method to screen NaN_3_ *in vitro* mutagenesis of *Plumbago indica* L. mutants at the seedling stage.

### 4.3 Accumulation of plumbagin content in *Plumbago indica* L. mutants

Previous studies have pointed out that a biologically active secondary metabolite, plumbagin, isolated from Plumbaginaceae plant, clearly showed anticancer potential both *in vitro* and *in vivo*(Liu et al., 2017b). Medicinal plants have a special function to produce a large number of bioactive substances in response to biotic or abiotic stresses, which are not only involved in plant growth, development or reproduction, but also enhance plant resistance (Singh et al., 2020). In our study, the accumulation of plumbagin content in the stems and leaves of *Plumbago indica* L. mutants were almost unaffected or even higher than that of CK, which could be that mutagenesis was an abiotic stress for *Plumbago indica* L., and responded to this stress. *Plumbago indica* L.retained or even enhanced this property, which was consistent with the results of previous studies. In addition, it had also been suggested that enhanced antioxidant enzyme activity, expression of resistance genes, and production of defense signaling molecules could stimulate the production and accumulation of secondary metabolites (Katoch et al., 2022), and in this study we screened cold-resistant mutants, and the original accumulation of plumbagin in the cold- resistant *Plumbago indica* L. mutants was greater than or equal to that of the control, suggesting that this the cold-resistant mutant, in addition to better mobilization of antioxidant enzyme activity, may also derive from its higher plumbagin content, which has better cold tolerance.

### 4.4 Relationship between anatomical and structural properties and cold resistance of *Plumbago indica* L. mutants

Mutagenesis causes morphological changes in plants (Li et al., 2021), and there are still limitations to the study of cellularity dissections. In ISSR polymorphism studies, it was found that NaN_3_ mutagenesis leads to a random point mutation of Hologene in *Plumbago indica* L. resulting in the deletion or increase of the DNA gene fragment may result in the acquisition of mutants with specific functions (Mago et al., 2017). Janosevic et al. (2011) also found leaf development in *Arabidopsis* mutants that the mesophyll cell became loose and the cellular layers were irregularly arranged, among other phenomena. In this study, the microstructure of the nine mutant plants screened also changed in varying degrees, with the more cold-tolerant mutants changing root, stem, and leaf microanatomy in response to cold stress. Sun et al. (2017) found in the anatomical feature of sugarcane roots after cold stress that the root cells gradually expanded, the cell walls started to break down and the structure started to be disordered. In cold-tolerant strains of *Plumbago indica* L., the cell of the root became larger and the thickness of xylem, phloem and cambium changed, which is consistent with the result that mutant strains have different cold resistance. Meanwhile, in a study on cold domestication of *rhododendron*, it was demonstrated that the ability of plants to acquire resistance to cold was correlated with lignin content (Liu et al., 2022). Unfortunately, although changes in the xylem in roots, stems, and leaves were found in this study, the lignin content and the size of the area occupied by the xylem were not examined, and relevant studies will be conducted in the next step.

Plant size reduction due to low temperature is a common phenomenon in temperate species and the phenomenon is considered to be an adaptive value of the plant to the cold environment (Lorenzo et al., 2019). At the same time, mutagenesis can cause dwarf due to damage to cells (Li et al., 2022c). The cold-resistant mutant strain screened in this case was also found to have dwarfed plants, which will be an important shape for future field performance to follow. Based on plant variation, cold environmental conditions lead to longer leaf epidermal cell margins, increased leaf thickness, and smaller leaves (Lorenzo et al., 2019). In turn, the increase in leaf thickness was correlated with the increase in the size of the mesophyll cell (Wang et al., 2008) or the number of mesophyll cell layers (Dumlao et al., 2012). We found a significant increase in leaf thickness of SA15 and SA24, which is consistent with the results of previous studies, so it is inferred that the level of cold resistance is positively correlated with leaf thickness. However, no significant changes were observed in SA19 and SA23, which were finally screened in this study, also indicating that plants acquire cold resistance as a complex process of multiple changes, and a single index cannot accurately determine their cold tolerance.

At the same time, cold stress tends to alter intracellular osmotic substances, resulting in leaf palisade tissue cellular structure that can be loosened by water loss, and SA19 in the study had the same cytological manifestation (Xu et al., 2022). In general, an increase in leaf thickness usually corresponds to an increase in palisade thickness, which is accompanied by an increase in leaf light driving capacity (Coble and Cavaleri, 2017). Thicker palisade tissues contain more chloroplasts to maximize light absorption, and there is growing evidence that f palisade tissues and spongy tissues are the main limiting factors for optimal photosynthesis, as evidenced by an increase in CTR with decreasing temperature and a gradual decrease in SR (He et al., 2018). In our study, we found an increase in CTR except for SA20, SA23, and SA24, indicating that the plant’s protective strategy when encountering cold damage tends to increase leaf photosynthetic capacity, which is another reason why cold-tolerant strains of *Plumbago indica* L. are resistant to cold. SA23 and SA24 were not increased, which may be related to the specificity of the mutant. In addition, SR values were found in the study and increased in different mutant strains, this reason is most likely due to the specificity exhibited by *Plumbago indica* L. being an herbaceous plant with high water use efficiency (He et al., 2018).

### 4.5 Physiological and ornamental traits assessment of cold tolerance in cold-tolerant mutants of *Plumbago indica* L.

When exposed to cold stress, plants naturally create a defense system. This process involves a number of physiological and biochemical changes, such as maintaining altered photosynthesis, maintaining ROS homeostasis, and altering the structure of the soluble sugar plasma membrane (Zhu, 2016; Zheng et al., 2022). Photosynthesis is an important physiological process in plants that is highly sensitive to changes in temperature, and Fv/Fm is an important parameter indicating plant exposure to cold stress (Yamori et al., 2014). In the present study, cold stress significantly reduced Fv/Fm, and these results are consistent with previous reports that cold stress reduces Fv/Fm. Second, cold stress may promote oxidative stress by inducing excessive production of ROS, while greatly reducing photosynthetic activity(Li et al., 2018b), when MDA levels and hydrogen peroxide levels are significantly elevated and cold damage occurs in the plant body, which is consistent with the results of the present study. Our study also found that the cold-tolerant mutant strains SA15, SA19, SA23, and SA24 had reduced relative MDA content, and increased SOD, CAT and POD activities, and were therefore more cold-tolerant, which also indicated that plants would mobilize different levels of antioxidant enzyme activity in the face of abiotic stress to respond to or alleviate injury (Li et al., 2022b; Duan et al., 2023). In addition, peroxidase plays a key role in cell elongation and cell wall thickening (Lorenzo et al., 2019), and the thickening of leaves and the thickening of epidermal cells found in this study all show the functional significance of mutants with stronger production of peroxidase to eliminate peroxide damage.

Sugar can also act as an inducer of signals to affect photosynthesis in response to low temperatures in winter (Liu et al., 2017a). The accumulation of sugars by plants in response to cold injury is also an important factor affecting cell structure, especially the cell wall and the mesophyll cell (Bilska-Kos et al., 2018). Sugars accumulate strongly in response to cold stress, polysaccharide was catabolized into soluble sugars and different types of sugars were accumulated to regulate osmotic potential depending on the severity and nature of the stress (Zheng et al., 2016). Proline, which has been widely reported to play an important role in cold stress or cold domestication for protection from cold injury, is considered another tolerance mechanism for abiotic stresses (Shin et al., 2018). The results of this study showed that the significant accumulation of soluble sugars and proline in the cold-tolerant strains SA15, SA19, SA23, and SA24 reflects one of the mechanisms to obtain higher tolerance from the physiological level. Therefore, combining anatomical features and physiological and biochemical indices, is a proven method to comprehensive screen for cold-tolerant mutants of *Plumbago indica* L.

In the field, the cold-tolerant mutants SA19 and SA24 were found to have reduced plant height, delayed flowering and short inflorescences, which are consistent with the studies of Li et al. (2022c) and Li et al. (2021). The phenomenon may be related to plant cell damage or DNA mutation due to mutagenesis, resulting in damage during plant growth and development, but such damage does not necessarily affect the ornamental traits of the plant (Amirikhah et al., 2019). In this study, the *Plumbago indica* L. cold-resistant mutant showed overall delayed flowering and delayed senescence, and was able to maintain good ornamental traits, making it a better mutant for both cold tolerance and medicinal ornamental properties.

## 5 Conclusion

**Figure 17:**
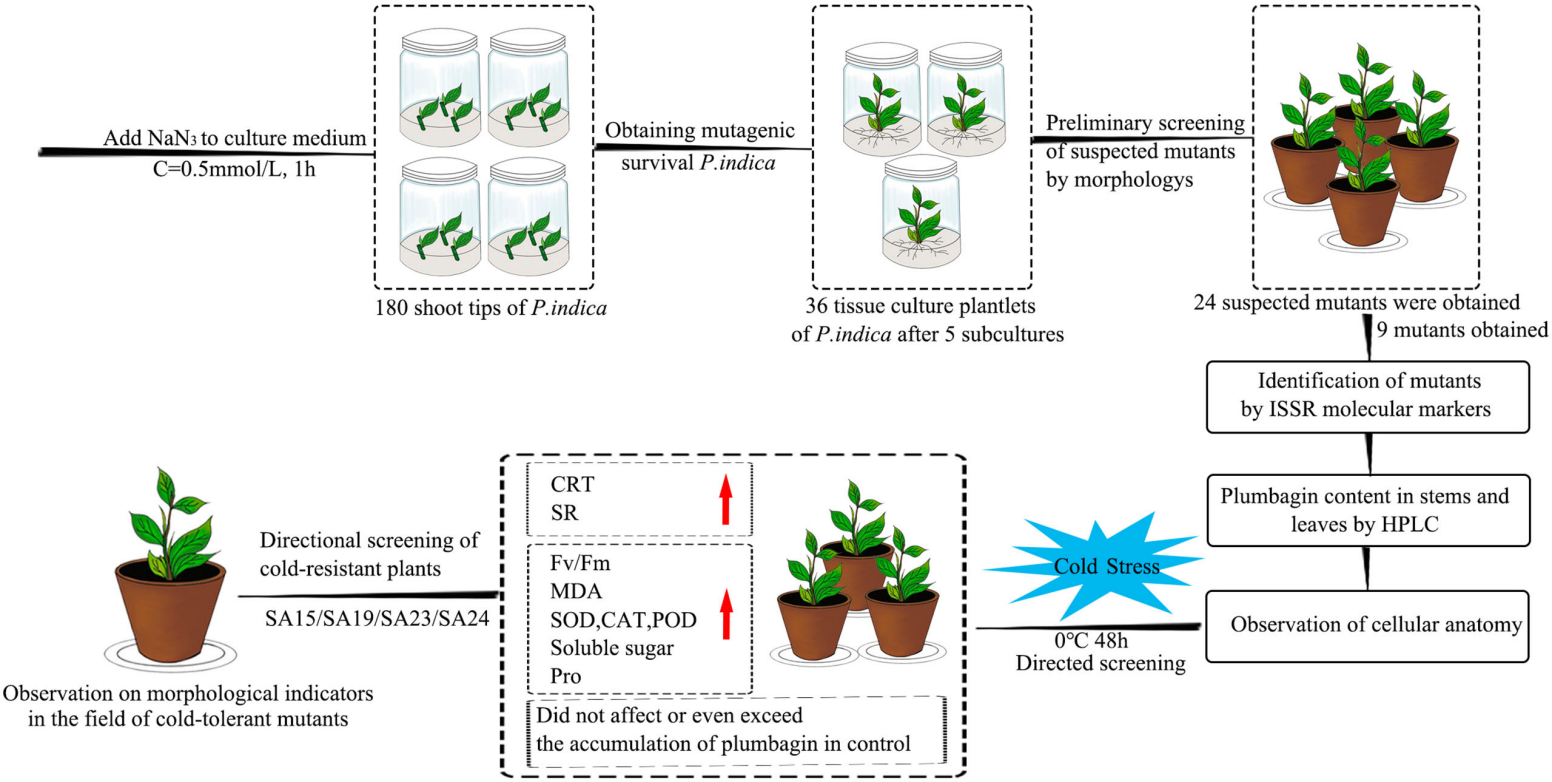
Flowchart for targeted screening of cold-tolerant mutants and evaluation of cold-tolerance in *Plumbago indica* L.

In this study, four cold-tolerant mutants were targeted and screened by a combination of ISSR molecular markers, morphological anatomy, and artificial low-temperature stress experiments using NaN_3_ mutagenesis treatment of *Plumbago indica* L., and their cold-tolerance characteristics were reported for the first time, and the technical procedure is shown (**Figure 17**). The mutagenic effect of NaN_3_ on the genetic variation occurring in the same genotype of *Plumbago indica* L.was remarkable, with 87.5% molecular marker polymorphism and 5.00% mutagenic efficiency of mutants. The four cold-tolerant mutants showed consistency in morphological and physiological and biochemical characteristics and initially showed the ability to adapt to short-term stress at low temperature of 0°C for 48h using ISSR markers combined with plumbagin content and morphological anatomical structures identification has a high accuracy in identifying *Plumbago indica* L. mutants.

## Data availability statement

The original contributions presented in the study are included in the article/**Supplementary Material**. Further inquiries can be directed to the corresponding author.

## Author contributions

XC and YL performed the experiments, and analyzed experimental data. JL and YZ participated in some data analysis. TL, LY, JNL and XY contributed by collecting some experimental materials. XC and SG designed the experiments. YL drafted the manuscript and supervised the project. SG supervised the project. The authors take responsibility for all aspects of the reliability and freedom from bias of the data presented and their interpretation. All authors have read and approved the final manuscript.
