# Supplementary material for: ISSR molecular markers and anatomical structures can assist in rapid and directional screening of cold-tolerant seedling mutants of medicinal and ornamental plant in Plumbago indica L
Source: Front Plant Sci. 2023 Jul 3;14:1149669. doi: 10.3389/fpls.2023.1149669 (PMC10350533; doi:10.3389/fpls.2023.1149669)
Supplement: Supplementary file 1 [file Table_1.docx]

Initialism

| CK | control check |
| --- | --- |
| CTAB | Cetyltrimethylammonium Bromide |
| CTR | Cell tense ratio |
| DNA | Deoxyribonucleic acid |
| FAA | FAA Fixative Solution |
| ISSR | Inter-simple sequence repeat |
| LE | Lower epidermal |
| LT | Leaf thickness |
| MDA | Malondialdehyde |
| MS | MS culture medium |
| PBS | Phosphate buffer solution |
| PCR | Polymerase Chain Reaction |
| RD | Root diameter |
| Pro | Proline |
| PT | Palisade tissue |
| SD | Stem diameter |
| SE | Stem epidermal cells |
| SR | Spongy ratio |
| ST | Spongy tissue |
| TBA | Thiobarbituric acid |
| TV | Thickness of main vein |
| UBC | University of British Columbia |
| UR | Upper epidermal |
